# Effects of Smoking on COVID-19 Management and Mortality: An Umbrella Review

**DOI:** 10.1155/2023/7656135

**Published:** 2023-05-13

**Authors:** SeyedAhmad SeyedAlinaghi, Amir Masoud Afsahi, Ramin Shahidi, Shaghayegh Kianzad, Zahra Pashaei, Maryam Mirahmad, Pooria Asili, Hengameh Mojdeganlou, Armin Razi, Paniz Mojdeganlou, Iman Amiri Fard, Sara Mahdiabadi, Arian Afzalian, Mohsen Dashti, Afsaneh Ghasemzadeh, Zohal Parmoon, Hajar Badri, Esmaeil Mehraeen, Daniel Hackett

**Affiliations:** ^1^Iranian Research Center for HIV/AIDS, Iranian Institute for Reduction of High Risk Behaviors, Tehran University of Medical Sciences, Tehran, Iran; ^2^Department of Radiology, School of Medicine, University of California, San Diego (UCSD), CA, USA; ^3^School of Medicine, Bushehr University of Medical Sciences, Bushehr, Iran; ^4^School of Medicine, Iran University of Medical Sciences, Tehran, Iran; ^5^Endocrinology and Metabolism Research Center, Endocrinology and Metabolism Research Institute, Tehran University of Medical Sciences, Tehran, Iran; ^6^Department of Pathology, Tehran University of Medical Sciences, Tehran, Iran; ^7^Department of Pathology, The Johns Hopkins University School of Medicine, Baltimore, MD, USA; ^8^School of Medicine, Tehran University of Medical Sciences, Tehran, Iran; ^9^Shahid Beheshti University of Medical Sciences, Tehran, Iran; ^10^Department of Community Health Nursing and Geriatric Nursing, School of Nursing and Midwifery, Iran University of Medical Sciences, Tehran, Iran; ^11^Department of Radiology, Tabriz University of Medical Sciences, Tabriz, Iran; ^12^School of Health, Guilan University of Medical Sciences, Rasht, Iran; ^13^Department of Health Information Technology, Khalkhal University of Medical Sciences, Khalkhal, Iran; ^14^Physical Activity, Lifestyle, Ageing and Wellbeing Faculty Research Group, School of Health Sciences, Faculty of Medicine and Health, The University of Sydney, Sydney, New South Wales, Australia

## Abstract

**Introduction:**

Smoking status appears to lead to a poor prognosis in COVID-19 patients. However, findings from the studies conducted on this topic have not been consistent, and further exploration is required.

**Methods:**

The objective of this umbrella review was to examine the effects of smoking on COVID-19 management and mortality. Online databases that included PubMed, Embase, Scopus, and Web of Science were searched using relevant keywords up to July 27, 2022. Articles were restricted to the English language, and the PRISMA protocol was followed.

**Results:**

A total of 27 systematic reviews, published from 2020 to 2022, were included. Individual studies included in the systematic reviews ranged from 8 to 186, with various population sizes. The consensus from the majority of systematic reviews was that COVID-19 smoker patients experience greater disease severity, disease progression, hospitalization rate, hospital admission duration, mechanical ventilation, ICU admission, and mortality rate.

**Conclusions:**

COVID-19 patients with a history of smoking (current and former) are vulnerable to adverse hospital outcomes and worse COVID-19 progression. Effective preventive and supportive approaches are required to decrease the risk of COVID-19 morbidity and mortality in patients with a history of smoking.

## 1. Introduction

Severe Acute Respiratory Syndrome Coronavirus-2 (SARS-CoV-2)—the cause of coronavirus disease 2019 (COVID-19)—first emerged in December 2019 in Wuhan, China [1–4]. The World Health Organization (WHO) declared a global COVID-19 pandemic in March 2020 [5]. As of August 8^th^, 2022, COVID-19 has infected more than 581 million people and caused more than 6.4 million deaths worldwide [6]. SARS-Cov-2 is mainly an aerosol-born disease and infects new cases through respiratory droplet inhalation [7–9]. COVID-19 presents mostly with flu-like symptoms such as fever, chills, myalgia, dry cough, fatigue, back pain, headache, anorexia, diarrhea, anosmia (loss of smell sensation), and ageusia (loss of taste sensation) [10, 11]. However, 4–41% of total cases can be asymptomatic [10, 11]. Severe COVID-19 can cause acute respiratory distress syndrome (ARDS), which manifests with hypoxia, dyspnea, chest pain, altered consciousness, cyanosis, and eventual death. It mainly occurs among older adults (i.e., >65 years) and vulnerable populations including patients with chronic kidney, liver, or lung disease, diabetes mellitus, obesity, HIV infection, and smoking history [12].

Smokers are more predisposed to viral and bacterial pulmonary infections including influenza, tuberculosis, and bacterial pneumonia [13–16]. In the first months of the pandemic in 2020, it was reported that Chinese patients with severe COVID-19 were mostly COPD patients or current smokers [17]. In addition, one of the earliest systematic reviews concluded that smoking is negatively associated with COVID-19 progression and prognosis [18]. Some studies have reported higher expression of angiotensin-converting enzyme 2 (ACE-2), which is the main receptor of SARS-CoV-2, in the lower respiratory airways of current smokers and COPD patients compared to nonsmokers, and stated that smoking and COPD could contribute to a higher COVID-19 incidence and relatively poorer outcomes [19, 20]. Conversely, some studies have reported lower levels of ACE-2 among smokers compared to nonsmokers [21, 22], and one preliminary meta-analysis of five studies in China stated smoking may not be significantly associated with an increased risk of severe disease among COVID-19 patients [23].

There have been five other meta-analyses conducted where the findings support the hypothesis that COPD and current smoking status contribute to worse progression and poor outcomes among COVID-19 patients [24–28]. One of the meta-analyses specifically stated that smoking can have negative adverse impacts on disease severity and mortality among hospitalized COVID-19 patients, with more impact on nondiabetic younger patients [27]. In addition, another meta-analysis specified that both current smoking and previous history of smoking increase COVID-19 severity significantly, while previous history of smoking increases the mortality risk [28]. Now that almost three years has passed since the first report of SARS-CoV-2 in human population, and large sample-sized studies and more reliable data are available, further investigation of COVID-19 and smoking is warranted. The aim of this review was to examine the associations between COVID-19 and smoking status (current smoker or history of smoking), answer the controversial paradoxes, and fill the gaps in the literature.

## 2. Methods

The objective of this umbrella study was to explore the prevailing systematic review literature pertaining the associations between COVID-19 and smoking status (being a current smoker or having history of smoking) and effects of smoking on COVID-19 management and mortality. In order to substantiate the results, the Preferred Reporting Items for Systematic Reviews and Meta-Analyses (PRISMA) checklist was followed. Quality of studies was evaluated with the NIH quality assessment tool.

### 2.1. Data Sources

An extensive search of four online databases was performed which included PubMed, Scopus, Embase, and Web of Science as data sources. Articles were restricted to the English language, and the search was conducted up to July 27, 2022. The following keywords and their combinations were used during the search:

- “COVID-19” OR “Novel coronavirus” OR “2019-nCoV” OR “SARS-CoV-2” OR “SARS- CoV2” [Title/Abstract]
- “Smoking” [Title/Abstract]
- “Systematic review” [Title/Abstract]
- [A] AND [B] AND [C]

### 2.2. Study Selection

In order to improve the study selection process, a two-step method was employed. The first step consisted of screening literature with regard to titles and abstracts. This was done by five researchers. The second step was performed by another five researchers, involving screening of full texts that were potentially eligible. Articles that met the inclusion criteria were advanced to the next step of data extraction. Articles were included if they had a systematic review nature and were peer-reviewed report on smoking and COVID-19. The exclusion criteria included studies lacking published data investigations, nonhuman research studies, duplications, abstracts with deficient full texts, editorial letters, conference abstracts, case series, and case reports.

### 2.3. Data Extraction

Data of publications having met the eligibility criteria and passing the second step of selection process was meticulously extracted and gathered in Table 1. Five researchers investigated the full texts and extracted these study requisites. Any duplicates were removed, and the accuracy of the extracted data was checked.

### 2.4. Quality and Risk of Bias Assessment

Study quality and risk of bias was assessed with the National Institute of Health (NIH) Quality Assessment (QA) Tools for Case Series Studies. Two independent reviewers rated the quality of the included studies.  Table 2 shows the results of the study quality and risk of bias. The scoring strategy of this tool has been explained at the bottom of this table.

## 3. Results

**Figure 1:**
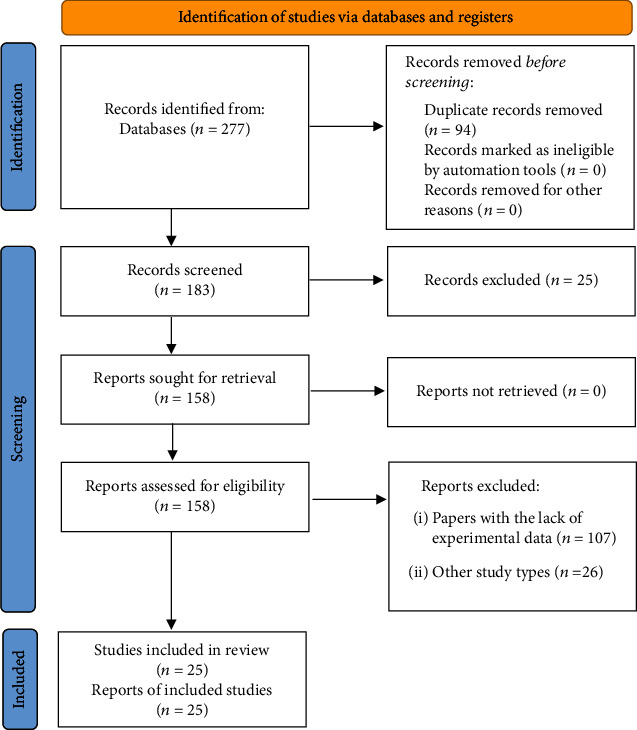
PRISMA 2020 flow diagram of study retrieval process.

The database search yielded 113 potential studies (after duplicates removed), and following the screening, a total of 27 articles met the eligibility criteria (Figure 1). The included systematic reviews were published between 2020 and 2022. One study included was designed as reviews of reviews [33].

Included studies were from China, the USA, Spain, Greece, Thailand, Canada, Indonesia, Japan, and other countries. China (7 studies) and the USA (5 studies) were the countries most represented. The 27 systematic reviews included a wide range of studies (i.e., 8 to 186 studies) with different populations (from 17 to 1,304,587 patients among mentioned populations). Males were the dominant population in 6 studies [29, 32, 37, 48, 49]. Table 1 shows the characteristics of the studies.

Smoking history was categorized as smokers and nonsmokers in some studies [46, 49], while most studies evaluated the smoking status as current and former smokers with comparisons between these groups [18, 40–42, 47]. One study represented smokers based on smoking heaviness, lifetime smoking, and smoking initiation [39].

Most of the studies concluded that smoker's with COVID-19 had a worse outcome and higher mortality rate [27, 30, 34, 36]. Evaluating the hospital admission rate, most of the studies showed increased risk of hospital admissions in smokers [18, 29, 30], while one study showed that smokers had lower risk of hospital admission [33].

The majority of studies found that smoking was associated with increased risk for mechanical ventilation [18, 29, 30]; however, there was one study showing no association between smoking status and risk of mechanical ventilation [51]. These results are shown in Table 3.

## 4. Discussions

According to the NIH's LitCovid database, more than 291,000 articles related to COVID-19 have been published to date, which shows the explosion of research in this field. This incredible amount of research has increased the information on different aspects of the disease. However, knowledge is still lacking in some areas with further research required to better understand COVID-19 and its effects. This umbrella review is aimed at organizing and updating the existing body of literature on the effects of smoking on COVID-19.

The overall findings support the hypothesis of increased severity of disease in COVID-19 smoker patients. Specifically, smoking is linked to more advanced COVID-19 outcomes, as manifested by the necessity for ICU admission, mechanical ventilation, and COVID-19-related death. Nevertheless, only a few studies demonstrated no significant link between smoking and COVID-19-related mortality [30] and mechanical ventilation [51]. Additionally, only one study found no link between smoking and an increased risk of death from COVID-19 [27]. Notably, a study in England, that investigated approximately 17 million patient documents, discovered that increased COVID-19-related mortality linked to smoking no longer remained significant after adjustment for the presence of preexisting chronic pulmonary disease. This suggests that smoking-induced comorbidities may be the cause of the overall death toll among COVID-19 smoker patients [53]. The results emphasize the need for further research elucidating the mechanisms by which smoking increases the incidence of unfavorable outcomes in COVID-19 patients.

Various potentially harmful compounds are present in tobacco products. Moreover, further chemicals are formed during aerosolization as a result of combustion or heating. Previous studies have demonstrated that pulmonary epithelium and vascular endothelium are both damaged by harmful compounds in cigarette smoke. The mucociliary clearance and epithelial barrier are compromised by damage to epithelial cells. Additionally, injured cells release modified molecules that activate specific lung receptors, activating acquired and innate immune responses [54]. Through a variety of mechanisms including the direct effect of nicotine, reperfusion injury after carbon monoxide-induced hypoxia, and particulate matter's abundance, tobacco smoke ingredients cause oxidative stress [55, 56].

The angiotensin II conversion enzyme-2 (ACE2) receptor, which is abundant in mucosal respiratory epithelial cells, has been associated with COVID-19 infection. It is probably the most reasonable explanation for the potential increased risk of death among smokers. Infection by the host-virus binding to the ACE2 receptors is likely a critical stage in SARS-CoV-2 infection [57–59]. Smokers have significantly higher levels of pulmonary ACE2 gene expression compared to nonsmokers [60]. Tobacco use can cause oxidative stress and inflammation in the lungs, making smokers more susceptible to bacterial or viral diseases [58, 61]. Oxidative stress reduces epithelial permeability, which may have substantial consequences for smokers with COVID-19 disease [62, 63]. Likewise, smoking leads to cardiovascular disease, chronic lung illness, diabetes, and other comorbidities that are related to worse outcomes in patients with COVID-19 infection [64].

On the contrary, there is evidence demonstrating that smoking may exert positive effects on COVID-19 disease severity, mediated by nicotine [51]. It is important to keep in mind that specific cigarette ingredients, like nicotine, may affect ACE2 differently from entire cigarettes [65]. The underlying mechanism might be attributed to the evidence showing nicotine might decrease tumor necrosis factor (TNF) expression in airway epithelial cells [66]. Moreover, nicotine may act as an agonist of the cholinergic anti-inflammatory pathway, which regulates the immune response and inflammatory reaction [67, 68].

Factors responsible for higher susceptibility of smokers to COVID-19 are briefly discussed here to better understand the mechanisms about how smoking can affect the human body:

- Smoking causes significant pathological changes including the mucosal epithelial barrier and an increase in the permeability of epithelial cells, which makes smokers more prone to be defeated against the virus invasion [69]
- Angiotensin-converting enzyme 2 (ACE 2) is defined as the principal receptor for SARS-CoV-2 virus to enter the host cell. ACE 2 expression is upregulated in the small airway epithelium of smokers, so the virus tends to invade host cells more easily. This upregulated receptor expression is one of the negative influences of oxidative stress due to smoking [20]
- Smoking can reduce the function of the immune system by causing a reduction in the number of CD4 + T cell (also named T helper cell, which activates macrophage or B cell), inhibiting the production of interleukin-22 (which moderates lung inflammation) and also, by promoting the secretion of catecholamines (which will weaken the immune system) [70, 71]
- The most serious complication of COVID-19 disease is acute respiratory distress syndrome (ARDS), as a result of the cytokine storm. In this situation, large amounts of proinflammatory cytokines and chemokines such as IP-10, IL-6, TNF-*α*, IFN-*γ*, IL-2, IL-7, and GM-CSF are released and entail severe immune system response which eventually causes lung inflammation and damage. In former smokers, the expression of IL-6, TNF-*α*, and other proinflammatory factors are increased [72]

Although most of the reviewed articles support the hypothesis that nicotine, the main component of cigarettes, increases the odds of developing severe illness of COVID-19, it is unknown whether the harm is related to nicotine or other toxic ingredients in cigarettes. Even some articles indicate that nicotine may have anti-inflammatory effects. Nicotine has been found to prevent acute lung damage and restrain the expression of tumor necrosis factor (TNF), which plays role in the inflammatory response [66]. Furthermore, nicotine is an agonist of the cholinergic anti-inflammatory pathway that modulates immune and inflammatory reaction [51, 73].

There have been a few original research studies comparing former and current smokers. The lung may heal when someone quits smoking, which may bias the findings if former smokers are part of the exposed group since one study found that the death rate among former smokers reduces with age [44, 45]. As a result, the frequency of smokers may have been underestimated, and some former smokers may have been incorrectly classified as nonsmokers [51]. As a result, only a few systematic reviews compared the features of these two groups. Three studies found that current smokers had lower risks of an adverse outcome than former smokers [31, 35, 51]. This might be because former smokers are more likely to be older, smoked for a longer period than current smokers, or because they have concomitant conditions such as asthma or COPD as a result of smoking [51]. Another factor might be that current smokers reported their status less than former smokers. [50], although a study found that current smokers had a greater likelihood of adverse outcomes than former smokers and nonsmokers [47].

Overall, compared with nonsmokers, smokers have higher risks of severe forms of the disease and hospitalization, and mortality [18, 39, 74]. On the contrary, the severity of COVID-19 was not associated with current or former smoking but with the comorbidities caused by smoking [75, 76]. In this study, the incidence of infection by SARS CoV-2 virus in smokers and nonsmokers cannot be evaluated clearly due to the lack of data at this present time.

## 5. Conclusion

There is strong evidence that smoking increases the risks of disease severity/progression, hospitalization, and mortality among COVID-19 patients. Encouraging smokers to quit using, early initiation of treatment after the onset of symptoms, timely vaccination, and promoting other preventive behaviors by public health providers can control the possibility of these people getting the infection and a better prognosis. Vaccination of smokers should be done completely among the priority groups.

## Figures and Tables

**Table 1:** Characteristics of studied items in the included papers.

First author (reference)	Country	Year of publication	Type of study	Population	Age	Gender	Smoking history
Alghamdi, S.A. [29]	Saudi Arabia	2020	Systematic review and meta-analysis	146,793 patients (from 11 studies)	55.2 y (mean age)	Male:55.4%	Smokers: 11,973 Nonsmokers: 134,820
Baker, J [30]	USA	2022	Systematic review	39 studies (populations varied from 101 to 406,793)	N/A	N/A	Smokers were ranging from 1.95% to 81.1% of populations
Farsalinos, K [31]	Greece	2020	Systematic review and meta-analysis	6,515 patients (18 published studies) 10,631 patients (prepublications)	N/A	N/A	440 smokers (out of 6115 pts) 961 smokers (out of 10631 pts)
Gonzalez-Rubio, J [32]	Spain	2020	Systematic review and meta-analysis	5,023 pts (18 studies)	N/A	53.2% male	Current smokers: 7.7%
Grundy, E. J [33]	UK	2020	Review of reviews	Varies from 17 to 387,109 pts for each of the included studies in ^∗^ studies (8 studies)	N/A	N/A	Patients divided in smokers, ex-smokers, and nonsmokers groups
Gulsen, A [34]	Turkey	2020	Systematic review and meta-analysis	10,797 patients (16 studies)	Mean age range: 38 to 62.2 y	N/A	Average smoking prevalence: 8.4% Range: 3.6 to 19.9%
Hou, H [35]	China	2021	Systematic review and meta-analysis	863,313 (73 articles)	N/A	N/A	Current smokers and former smokers
Kang, S [36]	China	2021	Systematic reviews	7041 (21 studies)	N/A	N/A	14.0% (984) had a history of smoking
Karanasos, A [27]	Greece	2020	Systematic review and meta-analysis	6310 patients (18 studies)	N/A	N/A	N/A
Kumar, R [37]	China (PRC), USA, and Europe	2021	Systematic review and meta-analysis	12037 (19 studies)	Age ranged from 23 to 91 years	55.68% male 42.76% females	N/A
Li, J [38]	China	2021	Systematic review and meta-analysis	2445 patients (12 studies)	N/A	N/A	N/A
Luo, S [39]	China	2022	Systematic review of Mendelian randomization studies	50 studies	N/A	N/A	Smoking include smoking initiation, smoking heaviness, and lifetime smoking
Mahamat-Saleh, Y [40]	France	2021	Systematic reviews and meta-analyses	186 studies 1,304,587 patients (210 447 deaths) (186 studies)	N/A	N/A	Former and current smoker
Mattey-Mora, PP [41]	USA	2022	Systematic reviews	1,002,006 patients (188,597 hospitalized patients) (40 study)	Mean age 44–68.8 years (from 11 study) Mean age 41–66.1 (from 11 study) 18 study not mentioned on age	N/A	Current/past smoking
Mesas, AE [42]	Spain	2020	Systematic review and meta-analysis	51,225 patients from 60 studies	Mean age range: 40-73 year	N/A	Current smokers and nonsmokers
Minh, LHN [43]	Japan	2021	Systematic review and meta-analysis	62,949 patients from 148 studies	N/A	N/A	Current smoker, external smoker, and never smoker
Patanavanich, R [44]	USA	2021	Systematic review and meta-analysis	22,939 patients from 46 studies	N/A	N/A	Current smokers, ex-smokers, and nonsmokers
Patanavanich, R [45]	Thailand	2022	Systematic review and meta-analysis	35,193 patients from 34 articles	Mean age 63.5 years	N/A	Current smokers, former smokers, and never smokers
Plasencia-Urizarri, TM [46]	Cuba	2020	Systematic review and meta-analysis	99,817 patients from 13 studies	N/A	N/A	Smokers and nonsmokers
Pranata, R [47]	Indonesia	2020	Systematic review and meta-analysis	4603 patients from 21 studies	>17 years	N/A	Current smokers, former smokers, and nonsmokers
Reddy, RK [28]	UK	2020	Systematic review and meta-analysis	32 849 hospitalized COVID-19 patients from 47 studies	N/A	N/A	8417 (25.6%) patients with a smoking history, comprising 1501 current smokers, 5676 former smokers, and 1240 unspecified smokers
Sanchez-Ramirez, DC [48]	Canada	2020	Systematic review and meta-analysis	13,184 COVID-19 patients from 22 studies	N/A	55% males	Current smokers, former smokers, and nonsmokers
Taylor, EH [49]	South Africa	2021	Systematic review and meta-analysis	44,305 patients from 58 studies	Mean age of 61.8 (60.7–63.0) years	68.9% males	Smokers and nonsmokers
Umnuaypornlert, A [50]	Thailand	2021	Systematic review and meta-analysis	369287 patients from 40 studies	Mean age 54.10 years	N/A	Current smokers, former smokers, and nonsmokers
Vardavas, C.I [18]	USA	2020	Sys review	Ranged from 41 to 1099 (5 studies)	N/A	N/A	Current smokers, former smokers, and nonsmokers
Zhang, H [51]	USA, China	2021	Sys review and meta-analysis	517020 pts(109 studies)	N/A	N/A	Patients divided to current smokers, former smokers, and never smoking group
Zhang, T [52]	China	2020	Sys review, meta-analysis and metaregression	Varies from 21 to 476 (16 studies)	Mean or median age varies from 39 to70.7y	Male is the dominant sex in 11 studies	Current smokers, former smokers, and nonsmokers

**Table 2:** Quality ratings of included studies based on NIH quality assessment (QA) tool for case series studies.

First author (# of ref)	^∗^Question									Rating by reviewers	
	1	2	3	4	5	6	7	8	9	# 1	# 2
Alghamdi, S.A. [29]	Yes	Yes	N/A	Yes	N/A	Yes	N/A	Yes	Yes	Good	Good
Baker, J [30]	Yes	Yes	N/A	Yes	N/A	Yes	N/A	Yes	Yes	Good	Good
Farsalinos, K [31]	No	Yes	N/A	Yes	N/A	Yes	N/A	Yes	Yes	Good	Fair
Gonzalez-Rubio, J [32]	No	Yes	N/A	Yes	N/A	No	N/A	No	Yes	Fair	Poor
Grundy, E. J [33]	Yes	Yes	N/A	Yes	N/A	No	N/A	No	Yes	Fair	Fair
Gulsen, A [34]	Yes	Yes	N/A	Yes	N/A	Yes	N/A	Yes	Yes	Good	Good
Hou, H [35]	Yes	Yes	N/A	Yes	N/A	Yes	N/A	Yes	Yes	Good	Good
Kang, S [36]	No	No	N/A	Yes	N/A	No	N/A	Yes	Yes	Fair	Fair
Karanasos, A [27]	Yes	Yes	N/A	Yes	N/A	Yes	N/A	Yes	Yes	Good	Good
Kumar, R [37]	Yes	No	N/A	Yes	N/A	Yes	N/A	No	Yes	Fair	Good
Li, J [38]	No	Yes	N/A	No	N/A	No	N/A	Yes	No	Poor	Poor
Luo, S [39]	Yes	Yes	N/A	Yes	N/A	No	N/A	No	No	Fair	Poor
Mahamat-Saleh, Y [40]	Yes	Yes	N/A	Yes	N/A	Yes	N/A	No	Yes	Good	Good
Mattey-Mora, PP [41]	No	Yes	N/A	Yes	N/A	No	N/A	No	No	Poor	Fair
Mesas, AE [42]	Yes	Yes	N/A	Yes	N/A	No	N/A	Yes	Yes	Fair	Good
Minh, LHN [43]	Yes	Yes	N/A	Yes	N/A	Yes	N/A	Yes	Yes	Good	Good
Patanavanich, R [44]	Yes	Yes	N/A	Yes	N/A	Yes	N/A	Yes	Yes	Good	Good
Patanavanich, R [45]	Yes	Yes	N/A	Yes	N/A	Yes	N/A	Yes	Yes	Good	Good
Plasencia-Urizarri, TM [46]	Yes	No	N/A	Yes	N/A	Yes	N/A	Yes	No	Fair	Poor
Pranata, R [47]	Yes	No	N/A	Yes	N/A	No	N/A	Yes	Yes	Good	Good
Reddy, RK [28]	Yes	Yes	N/A	Yes	N/A	Yes	N/A	Yes	Yes	Good	Good
Sanchez-Ramirez, DC [48]	Yes	Yes	N/A	Yes	N/A	Yes	N/A	Yes	Yes	Good	Good
Taylor, EH [49]	No	Yes	N/A	Yes	N/A	No	N/A	Yes	Yes	Good	Fair
Umnuaypornlert, A [50]	Yes	Yes	N/A	Yes	N/A	Yes	N/A	Yes	Yes	Good	Good
Vardavas, C.I [18]	No	Yes	N/A	Yes	N/A	Yes	N/A	Yes	Yes	Good	Good
Zhang, H [51]	Yes	Yes	N/A	Yes	N/A	Yes	N/A	Yes	Yes	Good	Good
Zhang, T [52]	No	Yes	N/A	Yes	N/A	No	N/A	Yes	Yes	Fair	Good

Note: NIH: National Institutes of Health; CD: cannot determine; NR: not reported; NA: not applicable. ^∗^The NIH quality assessment tool for case series studies (https://www.nhlbi.nih.gov/health-topics/study-quality-assessment-tools) contains nine questions: 1 = Was the study question or objective clearly stated?, 2 = Was the study population clearly and fully described, including a case definition?, 3 = Were the cases consecutive?, 4 = Were the subjects comparable?, 5 = Was the intervention clearly described?, 6 = Were the outcome measures clearly defined, valid, reliable, and implemented consistently across all study participants?, 7 = Was the length of follow-up adequate?, 8 = Were the statistical methods well-described?, 9 = Were the results well-described? (source: National Heart, Lung, and Blood Institute; National Institutes of Health; U.S. Department of Health and Human Services) https://www.nhlbi.nih.gov/health-topics/study-quality-assessment-tools.

**Table 3:** Description of the findings reported in the eligible studies.

First author (reference)	Effect of smoking on COVID-19							Other findings
	Disease progression	Disease severity	Outcome	Hospital admission	Increase hospitalization	ICU admission	Mechanical ventilation	
Alghamdi, S.A. [29]	More in smokers	More in smokers	Increased mortality and worse overall outcome in smokers	More admission in smokers	N/A	Increased in smokers	More in smokers	Smokers are more prone to have a severe disease and increased mortality rate

Baker, J [30]	More in tobacco users	More in tobacco users	-Increased mortality in tobacco users (17 studies) -Decreased mortality (1 study) No association between mortality and tobacco use (14 studies)	Higher admission rate in tobacco use	Longer hospital stay in tobacco users	Higher in tobacco users	Increased risk in tobacco use	Adverse outcome and higher mortality rate and more severe disease found in tobacco users

Farsalinos, K [31]	N/A	N/A	Worse outcome in current smoker, but still less than former smokers	Interesting finding of lower admission rate between smokers	N/A	N/A	N/A	More negative outcome among current smoker but les probability of hospital admission But overall, smoking in not protective against COVID infection

Gonzalez-Rubio, J [32]	N/A	N/A	N/A	Lower in smokers	N/A	N/A	N/A	Lower hospital admission in smokers

Grundy, E. J [33]	Higher risk of progression in smokers	Sever disease in smokers	Adverse outcome in smokers	More in smokers	N/A	N/A	N/A	Smokers have greater risk of severe disease

Gulsen, A [34]	N/A	Sever disease in smokers	Increased mortality in smokers	N/A	N/A	Higher risk of ICU admission	N/A	Risk of severe COVID is increased with both current smoking and previous history of smoking

Hou, H [35]	N/A	N/A	Mortality is significantly higher in former smokers compared to current smokers	N/A	N/A	N/A	N/A	Significant increase in mortality rate in smokers(especially in former smokers compared to current smokers)

Kang, S [36]	Increase rate of disease progression	Smoking increase disease severity	Smoking increase mortality	N/A	Smoking increases hospitalized	N/A	N/A	N/A

Karanasos, A [27]	N/A	Increased severity	Increased mortality	N/A	Smoking increases the risk of hospitalized	N/A	N/A	Association of smoking with severity was not significant (10 studies with 4152 patients)

Kumar, R [37]	N/A	Significant association with severity	N/A	N/A	N/A	N/A	N/A	

Li, J [38]	N/A	Significantly associated with severe COVID-19	N/A	N/A	N/A	Significantly associated with severe (ICU) COVID-19	N/A	

Luo, S [39]	N/A	Strong associations with smoking	Strong associations with smoking	N/A	Strong associations with smoking	N/A	N/A	

Mahamat-Saleh, Y [40]	N/A	N/A	Increased absolute risk of death	The absolute risk of COVID-19 death increased by 7%	N/A	N/A	N/A	In this study show risk of death increase in ever smoker 28% current 29% and former 25%

Mattey-Mora, PP [41]	N/A	N/A	N/A	N/A	Smoking increases hospitalized	N/A	N/A	

Mesas, AE [42]	N/A	N/A	Higher in-hospital mortality risk in smokers	N/A	N/A	N/A	N/A	N/A

Minh, LHN [43]	More in smokers	Higher risk of severity in smokers	Higher death risk in smokers	N/A	N/A	N/A	N/A	N/A

Patanavanich, R [44]	Higher disease progression in those with a history of smoking	N/A	Increased risk of death from COVID-19	N/A	N/A	N/A	N/A	N/A

Patanavanich, R [45]	N/A	N/A	Higher risk of death in current and former smokers compared to never smokers	N/A	N/A	N/A	N/A	The risk for COVID-19 death in current smokers does not vary by age, but significantly drops by age in former smokers

Plasencia-Urizarri, TM [46]	N/A	Higher risk for severe clinical presentations in smokers	N/A	N/A	N/A	N/A	N/A	N/A

Pranata, R [47]	N/A	N/A	Increased risk of composite poor outcome in smoker	N/A	N/A	N/A	N/A	Current smokers were at higher risk of composite poor outcomes than former/nonsmokers

Reddy, RK [28]	Increased risk of disease progression in patients with a smoking history	Increased risk of severe COVID-19 in current smokers	Increased in-hospital mortality in patients with a smoking history	N/A	N/A	N/A	Increased need for mechanical ventilation in patients with a smoking history	N/A

Sanchez-Ramirez, DC [48]	N/A	N/A	Higher rate of severe outcome in current and former smokers	N/A	N/A	N/A	N/A	N/A

Taylor, EH [49]	N/A	N/A	Higher ICU mortality rate in smokers	N/A	N/A	N/A	N/A	N/A

Umnuaypornlert, A [50]	N/A	Increased risk of disease severity in current and former smokers	Increased mortality in current and former smokers	N/A	N/A	N/A	N/A	N/A

Vardavas, C.I [18]	Smokers are more prone to have negative progression and severe symptoms	Smokers have severe disease	Higher mortality rate and adverse outcome was seen in smokers	More in smokers	N/A	Higher risk of ICU admission in smoker group	Higher risk of MV in smokers	There is an association between smoking and higher mortality and adverse outcome

Zhang, H [51]	N/A	Smoking was associated with severe COVID	Smokers have higher mortality rate	N/A	N/A	Higher risk of ICU admission was seen among smokers	No relationship was found between MV and smoking	Severe disease was more associated with former smoking compared to current smoking

Zhang, T [52]	Former smokers have more symptoms and severe disease	Former smokers are more prevalent in severe COVID group	Higher mortality rate is seen in severe cases (including former smokers)	N/A	N/A	N/A	N/A	Smoking is among the risk factors for severe COVID infection and complications

## Data Availability

The authors stated that all information provided in this article could be shared.

## Additional Points

*Implications*. (i) There is strong evidence that smoking increases the risks of disease severity/progression, hospitalization, and mortality among COVID-19 patients. (ii) Encouraging smokers to quit using, early initiation of treatment after the onset of symptoms, timely vaccination, and promoting other preventive behaviors by public health providers can control the possibility of these people getting the infection and a better prognosis. (iii) Vaccination of smokers should be done completely among the priority groups.

## Supplementary Materials

Supplementary Materials Supplementary table 1: description of the findings reported in the eligible studies.

## References

[1] Davies D., McDougall A., Yoong W. (2022). COVID-19 vaccination during pregnancy: coverage and safety, a comment. *American Journal of Obstetrics and Gynecology*.

[2] Mehraeen E., Dadras O., Afsahi A. M. (2022). Vaccines for COVID-19: a systematic review of feasibility and effectiveness. *Infectious Disorders Drug Targets*.

[3] SeyedAlinaghi S., Karimi A., Mojdeganlou H. (2022). Minimum infective dose of severe acute respiratory syndrome coronavirus 2 based on the current evidence: a systematic review. *SAGE Open Medicine*.

[4] Oliaei S., SeyedAlinaghi S., Mehrtak M. (2021). The effects of hyperbaric oxygen therapy (HBOT) on coronavirus disease-2019 (COVID-19): a systematic review. *European Journal of Medical Research*.

[5] Mehraeen E., Salehi M. A., Behnezhad F., Moghaddam H. R., Seyed A. S. (2021). Transmission modes of COVID-19: a systematic review. *Infectious Disorders Drug Targets*.

[6] World Health Organization WHO coronavirus (COVID-19) dashboard. https://covid19.who.int/.

[7] Centers for Disease Control and Prevention Transmission. https://www.cdc.gov/coronavirus/2019-ncov/transmission/index.html.

[8] Mehraeen E., Najafi Z., Hayati B. (2022). Current treatments and therapeutic options for COVID-19 patients: a systematic review. *Infectious Disorders Drug Targets*.

[9] Seyed Alinaghi S., Karimi A., Barzegary A. (2022). Mucormycosis infection in patients with COVID-19: a systematic review. *Health Science Reports*.

[10] Centers for Disease Control and Prevention Symptoms of COVID-19. https://www.cdc.gov/coronavirus/2019-ncov/symptoms-testing/symptoms.html.

[11] Byambasuren O., Cardona M., Bell K., Clark J., McLaws M.-L., Glasziou P. (2020). Estimating the extent of asymptomatic COVID-19 and its potential for community transmission: systematic review and meta-analysis. *Official Journal of the Association of Medical Microbiology and Infectious Disease Canada*.

[12] Ramadori G. P. (2022). SARS-CoV-2-Infection (COVID-19): Clinical Course, Viral Acute Respiratory Distress Syndrome (ARDS) and Cause(s) of Death. *Medical Sciences*.

[13] Atto B., Eapen M. S., Sharma P. (2019). New therapeutic targets for the prevention of infectious acute exacerbations of COPD: role of epithelial adhesion molecules and inflammatory pathways. *Clinical Science*.

[14] Eapen M. S., Sharma P., Moodley Y. P., Hansbro P. M., Sohal S. S. (2019). Dysfunctional immunity and microbial adhesion molecules in smoking-induced pneumonia. *American Journal of Respiratory and Critical Care Medicine*.

[15] Eapen M. S., Sharma P., Sohal S. S. (2019). Mitochondrial dysfunction in macrophages: a key to defective bacterial phagocytosis in COPD. *European Respiratory Journal*.

[16] Tuder R. M., Yun J. H. (2008). It takes two to tango: cigarette smoke partners with viruses to promote emphysema. *The Journal of Clinical Investigation*.

[17] Guan W. J., Ni Z. Y., Hu Y. (2020). Clinical characteristics of coronavirus disease 2019 in China. *New England Journal of Medicine*.

[18] Vardavas C. I., Nikitara K. (2020). COVID-19 and smoking: a systematic review of the evidence. *Tobacco Induced Diseases*.

[19] Jacobs M., Van Eeckhoutte H. P., Wijnant S. R. (2020). Increased expression of ACE2, the SARS-CoV-2 entry receptor, in alveolar and bronchial epithelium of smokers and COPD subjects. *The European Respiratory Journal*.

[20] Leung J. M., Yang C. X., Tam A. (2020). ACE-2 expression in the small airway epithelia of smokers and COPD patients: implications for COVID-19. *The European Respiratory Journal*.

[21] Oakes J. M., Fuchs R. M., Gardner J. D., Lazartigues E., Yue X. (2018). Nicotine and the renin-angiotensin system. *American Journal of Physiology-Regulatory, Integrative and Comparative Physiology*.

[22] Wan Y., Shang J., Graham R., Baric R. S., Li F. (2020). Receptor recognition by the novel coronavirus from Wuhan: an analysis based on decade-long structural studies of SARS coronavirus. *Journal of Virology*.

[23] Lippi G., Henry B. M. (2020). Active smoking is not associated with severity of coronavirus disease 2019 (COVID-19). *European Journal of Internal Medicine*.

[24] Zhao Q., Meng M., Kumar R. (2020). The impact of COPD and smoking history on the severity of COVID-19: a systemic review and meta-analysis. *Journal of Medical Virology*.

[25] Zheng Z., Peng F., Xu B. (2020). Risk factors of critical & mortal COVID-19 cases: a systematic literature review and meta-analysis. *Journal of Infection*.

[26] Astuti P. A. S. (2020). COVID-19 pandemic: an opportunity to enhance tobacco control in Indonesia. *Nicotine & Tobacco Research*.

[27] Karanasos A., Aznaouridis K., Latsios G. (2020). Impact of smoking status on disease severity and mortality of hospitalized patients with COVID-19 infection: a systematic review and meta-analysis. *Nicotine & Tobacco Research*.

[28] Reddy R. K., Charles W. N., Sklavounos A., Dutt A., Seed P. T., Khajuria A. (2021). The effect of smoking on COVID-19 severity: a systematic review and meta-analysis. *Journal of Medical Virology*.

[29] Alghamdi S. A., Alahmari A. S., Bajari S. K. (2020). Smoking and severity of COVID-19 infection: a short systematic review and meta-analysis. *Annals of Medical and Health Sciences Research*.

[30] Baker J., Krishnan N., Abroms L. C., Berg C. J. (2022). The impact of tobacco use on COVID-19 outcomes: a systematic review. *Journal of Smoking Cessation*.

[31] Farsalinos K., Barbouni A., Poulas K., Polosa R., Caponnetto P., Niaura R. (2020). Current smoking, former smoking, and adverse outcome among hospitalized COVID-19 patients: a systematic review and meta-analysis. *Therapeutic Advances in Chronic Disease.*.

[32] González-Rubio J., Navarro-López C., López-Nájera E. (2020). A systematic review and meta-analysis of hospitalised current smokers and COVID-19. *International Journal of Environmental Research and Public Health*.

[33] Grundy E. J., Suddek T., Filippidis F. T., Majeed A., Coronini-Cronberg S. (2020). Smoking, SARS-CoV-2 and COVID-19: a review of reviews considering implications for public health policy and practice. *Tobacco Induced Diseases*.

[34] Gülsen A., Yigitbas B. A., Uslu B., Drömann D., Kilinc O. (2020). The effect of smoking on COVID-19 symptom severity: systematic review and meta-analysis. *Pulmonary Medicine*.

[35] Hou H., Li Y., Zhang P. (2021). Smoking is independently associated with an increased risk for COVID-19 mortality: a systematic review and meta-analysis based on adjusted effect estimates. *Nicotine & Tobacco Research*.

[36] Kang S., Gong X., Yuan Y. (2021). Association of smoking and cardiovascular disease with disease progression in COVID-19: a systematic review and meta-analysis. *Epidemiology and Infection*.

[37] Kumar R., Rai A. K., Phukan M. M. (2021). Accumulating impact of smoking and co-morbidities on severity and mortality of COVID-19 infection: a systematic review and meta-analysis. *Current Genomics*.

[38] Li J., He X., Yuan Y. (2021). Meta-analysis investigating the relationship between clinical features, outcomes, and severity of severe acute respiratory syndrome coronavirus 2 (SARS-CoV-2) pneumonia. *American Journal of Infection Control*.

[39] Luo S., Liang Y., Wong T. H. T., Schooling C. M., Au Yeung S. L. (2022). Identifying factors contributing to increased susceptibility to COVID-19 risk: a systematic review of Mendelian randomization studies. *International Journal of Epidemiology*.

[40] Mahamat-Saleh Y., Fiolet T., Rebeaud M. E. (2021). Diabetes, hypertension, body mass index, smoking and COVID-19-related mortality: a systematic review and meta-analysis of observational studies. *BMJ Open*.

[41] Mattey-Mora P. P., Begle C. A., Owusu C. K., Chen C., Parker M. A. (2022). Hospitalised versus outpatient COVID-19 patients' background characteristics and comorbidities: a systematic review and meta-analysis. *Reviews in Medical Virology*.

[42] Mesas A. E., Cavero-Redondo I., Álvarez-Bueno C. (2020). Predictors of in-hospital COVID-19 mortality: a comprehensive systematic review and meta-analysis exploring differences by age, sex and health conditions. *PloS One*.

[43] Minh L. H. N., Abozaid A. A. F., Ha N. X. (2021). Clinical and laboratory factors associated with coronavirus disease 2019 (Covid-19): a systematic review and meta-analysis. *Reviews in Medical Virology*.

[44] Patanavanich R., Glantz S. A. (2021). Smoking is associated with worse outcomes of COVID-19 particularly among younger adults: a systematic review and meta-analysis. *BMC Public Health*.

[45] Patanavanich R., Siripoon T., Amponnavarat S., Glantz S. A. (2023). Active smokers are at higher risk of COVID-19 death: a systematic review and meta-analysis. *Nicotine & Tobacco Research*.

[46] Plasencia-Urizarri T. M., Aguilera-Rodríguez R., Almaguer-Mederos L. E. (2020). Comorbidities and clinical severity of COVID-19: systematic review and meta-analysis. *Revista Habanera de Ciencias Medicas*.

[47] Pranata R., Soeroto A. Y., Huang I. (2020). Effect of chronic obstructive pulmonary disease and smoking on the outcome of COVID-19. *The International Journal of Tuberculosis and Lung Disease*.

[48] Sanchez-Ramirez D. C., Mackey D. (2020). Underlying respiratory diseases, specifically COPD, and smoking are associated with severe COVID-19 outcomes: a systematic review and meta-analysis. *Respiratory Medicine*.

[49] Taylor E. H., Marson E. J., Elhadi M. (2021). Factors associated with mortality in patients with COVID-19 admitted to intensive care: a systematic review and meta-analysis. *Anaesthesia*.

[50] Umnuaypornlert A., Kanchanasurakit S., Lucero-Prisno D. E., Saokaew S. (2021). Smoking and risk of negative outcomes among COVID-19 patients: a systematic review and meta-analysis. *Tobacco Induced Diseases*.

[51] Zhang H. M., Ma S. D., Han T. T. (2021). Association of smoking history with severe and critical outcomes in COVID-19 patients: a systemic review and meta-analysis. *European Journal of Integrative Medicine*.

[52] Zhang T., Huang W. S., Guan W. (2020). Risk factors and predictors associated with the severity of COVID-19 in China: a systematic review, meta-analysis, and meta-regression. *Journal of Thoracic Disease*.

[53] Williamson E. J., Walker A. J., Bhaskaran K. (2020). Factors associated with COVID-19-related death using OpenSAFELY. *Nature*.

[54] Nyunoya T., Mebratu Y., Contreras A., Delgado M., Chand H. S., Tesfaigzi Y. (2014). Molecular processes that drive cigarette smoke-induced epithelial cell fate of the lung. *American Journal of Respiratory Cell and Molecular Biology*.

[55] Qasim H., Alarabi A. B., Alzoubi K. H., Karim Z. A., Alshbool F. Z., Khasawneh F. T. (2019). The effects of hookah/waterpipe smoking on general health and the cardiovascular system. *Environmental Health and Preventive Medicine*.

[56] Piantadosi C. A. (2008). Carbon monoxide, reactive oxygen signaling, and oxidative stress. *Free Radical Biology & Medicine*.

[57] Arcavi L., Benowitz N. L. (2004). Cigarette smoking and infection. *Archives of Internal Medicine*.

[58] Bauer C. M. T., Morissette M. C., Stämpfli M. R. (2013). The influence of cigarette smoking on viral infections: translating bench science to impact COPD pathogenesis and acute exacerbations of COPD clinically. *Chest*.

[59] Strzelak A., Ratajczak A., Adamiec A., Feleszko W. (2018). Tobacco smoke induces and alters immune responses in the lung triggering inflammation, allergy, asthma and other lung diseases: a mechanistic review. *International Journal of Environmental Research and Public Health*.

[60] Cai G., Bossé Y., Xiao F., Kheradmand F., Amos C. I. (2020). Tobacco smoking increases the lung gene expression of ACE2, the receptor of SARS-CoV-2. *American Journal of Respiratory and Critical Care Medicine*.

[61] Yao H., Rahman I. (2011). Current concepts on oxidative/carbonyl stress, inflammation and epigenetics in pathogenesis of chronic obstructive pulmonary disease. *Toxicology and Applied Pharmacology*.

[62] Wiener R. S., Cao Y. X., Hinds A., Ramirez M. I., Williams M. C. (2007). Angiotensin converting enzyme 2 is primarily epithelial and is developmentally regulated in the mouse lung. *Journal of Cellular Biochemistry*.

[63] Meng Y., Li T., Zhou G. S. (2015). The angiotensin-converting enzyme 2/angiotensin (1–7)/mas axis protects against lung fibroblast migration and lung fibrosis by inhibiting the NOX4-derived ROS-mediated RhoA/rho kinase pathway. *Antioxidants & Redox Signaling*.

[64] Richardson S., Hirsch J. S., Narasimhan M. (2020). Presenting characteristics, comorbidities, and outcomes among 5700 patients hospitalized with COVID-19 in the New York City area. *Journal of the American Medical Association*.

[65] Ferrari M. F., Raizada M. K., Fior-Chadi D. R. (2008). Differential regulation of the renin-angiotensin system by nicotine in WKY and SHR glia. *Journal of Molecular Neuroscience*.

[66] Li Q., Zhou X. D., Kolosov V. P., Perelman J. M. (2011). Nicotine reduces TNF-*α* expression through a *α*7 nAChR/MyD88/NF-ĸB pathway in HBE16 airway epithelial cells. *Cellular Physiology and Biochemistry*.

[67] Wang H., Yu M., Ochani M. (2003). Nicotinic acetylcholine receptor *α*7 subunit is an essential regulator of inflammation. *Nature*.

[68] Tracey K. J. (2007). Physiology and immunology of the cholinergic antiinflammatory pathway. *The Journal of Clinical Investigation*.

[69] Aghapour M., Raee P., Moghaddam S. J., Hiemstra P. S., Heijink I. H. (2018). Airway epithelial barrier dysfunction in chronic obstructive pulmonary disease: role of cigarette smoke exposure. *American Journal of Respiratory Cell and Molecular Biology*.

[70] Nguyen H. M. H., Torres J. A., Agrawal S., Agrawal A. (2020). Nicotine impairs the response of lung epithelial cells to IL-22. *Mediators of inflammation*.

[71] Yue H. E., Jian S. U., Xiaoqian D. I., Qiang W. A. (2021). Mechanisms in which smoking increases the risk of covid-19 infection: a narrative review. *Iranian Journal of Public Health*.

[72] Li X., Geng M., Peng Y., Meng L., Lu S. (2020). Molecular immune pathogenesis and diagnosis of COVID-19. *Journal of Pharmaceutical Analysis*.

[73] Tracey K. J. (2007). Physiology and immunology of the cholinergic antiinfammatory pathway. *The Journal of clinical investigation*.

[74] Clift A. K., Von Ende A., San Tan P. (2022). Smoking and COVID-19 outcomes: an observational and Mendelian randomisation study using the UK Biobank cohort. *Thorax*.

[75] Matsushita Y., Yokoyama T., Hayakawa K. (2022). Smoking and severe illness in hospitalized COVID-19 patients in Japan. *International Journal of Epidemiology*.

[76] Korzeniowska A., Ręka G., Bilska M., Piecewicz-Szczęsna H. (2021). The smoker’s paradox during the COVID-19 pandemic? The influence of smoking and vaping on the incidence and course of SARS-CoV-2 virus infection as well as possibility of using nicotine in the treatment of COVID-19 - review of the literature. *Przegląd Epidemiologiczny*.

