# Supplementary material for: Effects of Smoking on COVID-19 Management and Mortality: An Umbrella Review
Source: J Smok Cessat. 2023 May 13;2023:7656135. doi: 10.1155/2023/7656135 (PMC10199802; doi:10.1155/2023/7656135)
Supplement: Supplementary Materials — Supplementary table 1: description of the findings reported in the eligible studies. [file 7656135.f1.docx]

**Supplementary table 1.** Description of the findings reported in the eligible studies

| **ID** | **First Author** | **Date**  **(MM/YY)** | **Type of study/ Searched databases** | **Included studies** | **Total**  **Population** | **Sex** | **Other**  **comorbidities** | **Mortality** | **Outcome** | **Summary of findings** |
| --- | --- | --- | --- | --- | --- | --- | --- | --- | --- | --- |
| 1 | Cooper, T.J. | Oct 2020 | Systematic review/ Scopus, EMBASE, Medline | 8 | 70 HIV-infected patients (13 with AIDS ,57 without AIDS) | -- | -- | ↔ | Symptoms of patients are similar to general population | -No risk of poorer COVID-19 in well controlled HIV  - risk of Super infection exists and it might lead to severe COVID-19 |
| 2 | Costenaro, P. | Sep 2020 | Systematic review/ EMBASE, Medline, Google Scholar | 23 | 164 adults with COVID-19 and HIV co-infection | 84.5% male (120)  -female:20  -transgender: 2 | 101 cases  - HTN: 35 patients  -Dyslipidemia:20  -Diabetes: 20  And IHD, COPD, Chronic kidney disease  Lymphoma, HBV co infection hepatocarcinoma, hypothyroidism, pulmonary TB,  -3 cases in intensive care and 3 cases among patients who died had comorbidities | ↔ | -16 cases died  -15 patients needed intensive care | - No clear evidence of severe COVID or higher rate of infection in HIV –infected patients  - Male HIV patients with long term antiretroviral therapy may have severe COVID course |
| 3 | Danwang, c. | Jan 2022 | Systematic review and Meta-analysis/ Pubmed, Scopus, EMBASE, Web of science | 44 | 38,971,065 | -- | -- | ↑ | NO increase in severity of COVID was seen in Co-infection | -Increased risk of hospital admission in HIV patients  -HIV does not increase the mortality and severity of COVID |
| 4 | De Medeiros, KS. | Jul 2021 | Systematic review/ Medline, Scopus, EMBASE, Web of science, CINAHL, LILACS, Cochrane Clinicaltrials.gov | 30 | 266 | 209 males  57 females | -cardiovascular disease  -Obesity  -HTN  -COPD | ↓ | no relationship was found | -HIV infection might stop COVID infection due to IFN-l induction  -ART might prevent COVID infection |
| 5 | Dolatian, M. | Oct  2020 | Systematic review/ Scopus, EMBASE, Web of science, PubMed, Google Scholar | 15 | -- | -- | -- | ↔ | Only CD4<200 cells may increase risk of COVID infection | No significant difference was found in mortality and clinical signs of COVID in HIV positive individuals |
| 6 | Dong, Y. | Jul 2021 | Meta-analysis/ EMBASE, PubMed, China national Knowledge infrastructure, WanFang data, Chongqing VIP | 10 | 18,122,370  COVID-19 patients (41,113  COVID patients with HIV  And 18,081 COVID patients without HIV) | -- | -- | ↑ | increase mortality | HIV/COVID co infection may lead to higher mortality risk of COVID infection |
| 7 | Gao, y. | Aug 2020 | Systematic review and  Meta-analysis/ PubMed, Web of science, EMBASE, Cochrane, China national Knowledge infrastructure (CNKI), Chinese biomedical literature Database, Wanfang  database | 8 | 4007 | Male:  2256  female: 1751 | -- | ↑ | leads to severe COVID-19 disease | Immunodeficiency is associated with severe COVID-19 |
| 8 | Heidary, M. | Feb 2022 | Systematic review/ PubMed, EMBASE, Scopus | 65 | -- | Male: 74.3% | Most common: HTN  Diabetes Mellitus  Others: HBV, HSV2, HCV, | ↑ | HIV might elevate the morbidity and mortality of COVID-19 | HIV/COVID co-infected patients have higher comorbidities (HTN, Diabetes) compared to COVID-19 individuals without HIV |
| 9 | Huang, D. | Mar  2022 | Systematic review/ PubMed, Web of science, Wanfang, CNKI, SinoMed | 18 | 412  HIV/COVID patients | Male:  72% | 37.5% of patients had comorbidities  -HTN (most common0  -Chronic lung disease | ↓ | HIV/COVID patients with earlier use of ART had better outcome | Comorbidities increased the risk of severe COVID-19 |
| 10 | Kouhpayeh, H | Dec 2021 | Meta-analysis/ PubMed, Scopus | 11 | 19,645,775 COVID-19 infected case,  59,980 HIV-positive | -- | HTN,  Diabetes  Cardiovascular disease | ↑ | COVID-19 cause higher mortality in HIV positive patients | Mortality in HIV/COVID co infection is 21% higher than individuals without HIV |
| 11 | Lee, K.W. | Mar  2021 | Systematic review and  Meta-analysis/ PubMed, Medline, CINAHL | 82 | 643,018 HIV patients | Female: 58.5%  Male:39.8%  Unknown Gender:1.5%  Transgender:0.2% | -Cardiovascular (27.2%) (HTN,..)  -Diabetes Mellitus (12.2%)  -chronic lung disease:4.2%  -Asthma: 4.2%  -dyslipidemia  -obesity  -alcoholism  -smoking  -Chronic kidney disease  -Liver disease | ↑ | Long-term HIV might be associate with more severe illness | Mortality rate is higher in HIV /COVID con infected patients. |
| 12 | Liang, M. | Sep 2021 | Systematic review and  Meta-analysis/ PubMed, EMBASE, MedRxiv, BioRxiv | 14 | 203,761  COVID-19 patients | -- | -HTN  -Diabetes Mellitus  -Chronic Cardiac and kidney disease | ↑ | Comorbidities are related to poorer outcome | increased mortality in HIV/COVID co infection was seen with comorbidities |
| 13 | Massarvva, T. | May 2021 | Systematic review/ EMBASE, Scopus, Medline, Cochrane | 22 | 730 COVID-19/HIV patients | Male: 79.4% | HTN | ↑ | Comorbidities in HIV/COVID Co-infected patients are associate with poorer outcome.  Covid-19 outcome is better when HIV is controlled | Comorbidities in HIV/COVID co-infection leads to high risk COVID infection |
| 14 | Mellor, M.M. | Mar 2021 | Systematic review/ EMBASE, Google Scholar, Medline, Medrxiv, Google, Litcovid, Trip | 19 | (Large number)  10 studies included more than 1000 individuals | -- | Diabetes  Obesity | ↑ | COVID/HIV co-infection leads to higher mortality rate | HIV infection is associated with higher mortality rate |
| 15 | Mirzaei, H. | Jan 2021 | Systematic review/ PubMed, Web of science, Scopus, Google Scholar, preprint database | 25 | 252 patients with HIV/COVID-19 co infection | Male: 80.9%  Female: 18.3%  Transgender women: 0.8% | HTN  Hyperlipidemia  Obesity  COPD  Diabetes | ↔ | Presence of comorbidities is associate with severe COVID/HIV co infection and higher mortality | No difference in severity of COVID /HIV co infection and mortality between male or females |
| 16 | Oyelade, T. | Feb 2022 | Systematic review and  Meta-analysis/ EMBASE, Medline | 43 | 692,032 COVID-19 individuals (9097 of them have HIV) | -- | -- | ↑ | COVID/HIV co-infection may lead to higher mortality risk | HIV infection might cause more severe COVID infection |
| 17 | Patel, R.H. | Mar 2021 | Systematic review/ Pubmed/Medline | 63 | 4259 | -- | Diabetes  HBV  HTN  Obesity  COPD  Hyperthyroidism | ↓ | Comorbidities are associate with a poor prognosis of COVID infection | -HIV patients on ART have a better COVID outcome |
| 18 | Sarkar, S. | Dec 2020 | Systematic review and  Meta-analysis/ EMBASE, Google Scholar, Pubmed/Medline, preprint platform MedRxiv | 20 | 205,702 patients  177,186 patients with COVID/HIV co infection | -- | -- | ↔ | HIV/COVID co infection does not show any increased mortality risk | No significant increased mortality risk in COVID/HIV co infection |
| 19 | SeyedAlinaghi, S. | Jul 2021 | Systematic review/ PubMed, Web of science, Scopus, Science direct | 36 | 3,993,400 COVID patients  (89,343 patients with COVID-19/HIV Co infection) | Male:72%  Transgender: 0.01% | -HTN  -Diabetes Mellitus  -Asthma  -Renal insufficiency  -Cardiovascular disease | ↓ | Individual with advanced stage of HIV and low CD4 count have less severe COVID symptoms and less mortality after COVID infection | Advanced stages of HIV associated with COVID infection lower mortality rate |
| 20 | Shareef, M.A. | Oct 2020 | Systematic review/ PubMed, Cochrane, Medline, WHO registry | 7 | 16 | Male: 15  Female: 1 | -HTN  -Diabetes Mellitus  -Obesity  -COPD  -CAD | ↓ | Symptoms of COVID in HIV patients are similar to individuals without COVID | -Older population with comorbidities has poorer outcome  -COVID/HIV co-infected individuals with antiretroviral therapy (ART) have favorable outcome |
| 21 | Ssentongo, P. | Mar 2021 | Systematic review and  Meta-analysis/ PubMed, Web of science, Scopus, Cochrane, Google Scholar, OVID, preprint platform MedRxiv | 22 | 20,982,498 | Male: 66 % | -HTN  -Diabetes Mellitus  -COPD  -Chronic kidney disease | ↑ | Mortality rate due to COVID is higher in HIV positive individuals | -Risk of COVID infection is higher in HIV positive individuals  - Vaccination is highly recommended in HIV patients |
| 22 | Tamuzi, J. L. | Oct 2020 | Systematic review/ PubMed, Cochrane, MedRxiv, Google Scholar, WHO COVID database | 21 | 28,387 COVID patients (1094 COVID/HIV/TB and 1 SARS-COV/HIV/TB patients) | -- | HIV/TB co infection | ↑ | HIV/TB co-infection with COVID has more higher mortality rate | COVID associated with HIV/TB co infection is more common in males and recovery takes longer. |
| 23 | Varshney, K. | Jan 2022 | Systematic review/ PubMed, Scopus, Global Health, WHO Corona virus database | 20 | -- | -- | -Cardiovascular disease  -Diabetes  -Obesity  Chronic kidney Disease | ↔ | Mortality is about 2 time is male compared to Female | -Older age is related to higher mortality rate  -comorbidities are associated with higher mortality  - lower CD4 cell count does not affect mortality |
| 24 | Wang, H. | Nov 2021 | Meta-analysis/PubMed, Web of science, MedRxiv | 9 | -- | -- | -Diabetes  HTN  Cardiovascular disease  -Chronic Kidney disease  -Respiratory disease | ↑ | Comorbidities lead to increased severity of COVID infection | -COVID/HIV co infection increased the severity of COVID infection  - comorbidities also cause experiencing severe COVID disease |
| 25 | Wang, Y. | Sep 2021 | Meta-analysis/ PubMed, Web of science, EMBASE | 84 | 816,678 | -- | -- | ↑ | HIV infection is related to increased mortality | -COVID/HIV co infected individuals has higher mortality risk |
